# Through a relational lens: reflections about foster care experience in Italian emancipated foster youth

**DOI:** 10.3389/fpsyg.2023.1191307

**Published:** 2023-07-18

**Authors:** Paola Cardinali, Fabiola Bizzi, Laura Migliorini

**Affiliations:** ^1^Department of Economics, Mercatorum University, Rome, Italy; ^2^Department of Education Sciences, University of Genoa, Genoa, Italy

**Keywords:** emancipated foster youth, qualitative design, relational outcome, emerging adulthood, Italian study

## Abstract

The study aimed to investigate relational outcomes of Italian emancipated foster youth across open-ended reflections about their perceptions of their relationships with the biological and foster family, with partner and peers. A total of 26 Italian emancipated foster youth (19–25 years old) recruited by social services completed a single in-depth interview. A qualitative thematic analysis was selected for this study. The results revealed two major themes of foster care experience that emerged often simultaneously from the participants’ narration: (1) Positive Relational Outcomes including “Feeling supported,” “Sense of belonging,” “Good memories,” “Relationship with family of choice,” and “Staying in touch”; and (2) Negative Relational Outcomes referring “Devaluation,” “Refuse,” “Bad memories,” and “Isolation.” Foster care experience leads to complex relational perceptions in emerging adulthood according to different relational outcomes and ways of processing foster care experience. Professionals could work on relational outcomes and memories, especially during a critical transition like emerging adulthood, to support youth in making sense of their past experiences.

## 1. Introduction

### 1.1. Emancipated foster youth and emerging adulthood

Every year, many young people leave the foster care and protection system, approaching adulthood. Although there are several projects to support the transition to adulthood through gradual emancipation processes, it is important to promote a reflection on the experience of foster care at this stage of life to understand which processes can support processes of well-being and resilience among transition-aged foster youth (Richardson and Yates, 2014). In the process of foster care, a delicate balance of the affects is experienced and in this relational specificity it can be difficult to evaluate the outcome (Miller and Collins-Camargo, 2016). However, the study of the outcomes of foster care takes on a specific value for the young adult because this phase of transition towards autonomy is crucial for care leavers for the definition of their identity, of future projects and the fundamental choices in the intimate relationships that the experiences related to foster care in childhood and adolescence have challenged. Resilience framework could represent a useful tool for framing how emancipated foster youths could successfully overcome their adversities (Masten, 2018; Hokanson et al., 2020).

Emancipated foster youth represent a specific group with cumulative disadvantage, they experience profound disruptions in relationships, and they traverse multiple placements in care and institutional transitions before their emerging adulthood. Literature highlights that foster youths experience difficulties during this time period (e.g., Dworsky and Courtney, 2010; Berzin et al., 2011; Hook and Courtney, 2011), yet little is known about how they conceive and experience this transition in relation to the characteristics of emerging adulthood (Berzin et al., 2014). This period defined as emerging adulthood, refers to a developmental period between the ages of 18 and 25 when youth explore and make important decisions for their lives. During this period youth might be more self-focused than in other times in their life, they commonly experience instability, identity exploration, optimism, and feeling in-between adolescence and adulthood (Arnett, 2004, 2015; Schwartz et al., 2011). Youths tend to have parents and other adults that assist them through this challenging period towards the emancipation, yet youth with out-of-home care backgrounds may lack the social capital helpful in this transition (Sulimani-Aidan and Melkman, 2018). Indeed, individuals who have come out of the residential care system because they have reached the age beyond which they can no longer benefit from the care, “the emancipated foster youth,” can be limited in experiencing supporting opportunities.

Some studies encouraged the development of programs that extend foster care benefits to youth who emancipated from the system (Curry and Abrams, 2014). The past experiences of loss, separation, and the possible rejection in their birth families, the precarious placements, and transitory relationships with social workers, and limited environmental resources could make this passage toward adulthood more critical (Sulimani-Aidan and Melkman, 2018; Driscoll, 2019). Consequently, these harsh experiences in childhood and adolescence can have an impact on the development of their relationships, and negatively affected their transition to adulthood (Greeson and Bowen, 2008; Tyrell and Yates, 2018). Therefore, a focus on the constructive experiences and healthy relationships of vulnerable populations (i.e., Munson et al., 2013), as among emancipated foster youth (Berzin et al., 2014; Tyrell and Yates, 2018) could improve the outcomes of emancipated foster youth promoting avenues for intervention during the sensitive period of developmental reorganization that characterizes the transition from adolescence to young adulthood (Greeson and Bowen, 2008; Tyrell and Yates, 2018).

### 1.2. Relational outcomes in emancipated foster youth

According to the international literature, researchers have described emancipated foster youth as a vulnerable population in various areas of adult life, detecting difficulties in education, employment, and risky behaviors (Vinnerljung and Hjern, 2011; Berzin et al., 2014), difficulties in developing and maintaining new attachments when they emancipate from the foster care system (Yates and Grey, 2012), and inabilities to develop trusting, supportive relationships in adulthood (Cooley et al., 2018). However, other researchers (Samrai et al., 2011; Singer et al., 2013; Frimpong-Manso, 2020) have argued that positive outcomes for emancipated foster youth depend on several factors such as networks of social support, personal capacities, preparation for adulthood, and positive relationships. This suggests that besides a vulnerability and adversity that youth frequently experience following aging out of foster care, positive relationships may buffer the negative outcomes succeeding emancipation (Greeson and Bowen, 2008). Foster research has emphasized the need for caring relationships to be maintained in the lives of individuals in the transition to adulthood, showing the beneficial effects of caring relationships such as the strengthening of identity, the reduction of feelings of loss and rejection, and the promotion of self-esteem (Delgado et al., 2019; Wissö et al., 2019; Ball et al., 2021). Alongside, relationships with the family of origin, with others, romantic relationships, as well as affective experiences play a key-role on positive outcomes of American emancipated foster youth (Tyrell and Yates, 2018). However, foster youth express that support from the child welfare system affects their ability to be independent and to establish themselves as adults in emerging adulthood (Berzin et al., 2014).

The studies focused on negative and positive outcomes in a foster care context mainly used a qualitative approach investigating the resilience process (Parry and Weatherhead, 2014; Anderson and Williams, 2018), academic outcomes (Rios and Rocco, 2014), subjective experiences of “aging out” of emancipated foster youth (e.g., economic challenges and housing instability, loss of social support, and pressure to be self-reliant; Cunningham and Diversi, 2013) and relational outcomes (Berzin et al., 2014; Tyrell and Yates, 2018). Little is known about Italian youth with an out-of-home care background context (Corradini, 2018). Among the few studies, Italian researchers mainly investigated the foster families functioning (Migliorini et al., 2016, 2018; Migliorini and Bizzi, 2019) and the cultural aspects of foreign children in a relatively recent immigration country such as Italy (Long and Ricucci, 2016; Grumi et al., 2017; Rania et al., 2018).

Relational outcomes in Italian emancipated foster youth are still an area unexplored. However, it is important to note that the last data available from 31 December 2019 in reference to the final report of the survey on foster care in Italy asserted that there were 13,555 children and adolescents (evenly distributed between boys and girls but with territorial differences) placed in foster families (Minister for Labour and Social Policy, 2021). Of these there is a substantial prevalence of pre-adolescents and adolescents that strongly poses the need to build adequate accompaniment towards paths of autonomy. However, the datum does not count unaccompanied foreign minors placed in foster care as persons who live the out-of-home experience for specific status of single persons on the territory and not because they are removed by the family with a measure ordered by the Tribunal for minors or the judge protect. In addition, every year, 3,000 emancipated foster youth leave their foster family, and about one-third of them return to their biological family. Little is known of them: long-term monitoring is difficult, much information continues to be lacking, as strong and unresolved discrepancies in the data are common, and outcome evaluation remains an under-explored area.

In order to overcome the gap in the literature related to the investigation of relational outcomes of emancipated foster youth in Italy, the current study drew on Italian newly emancipated foster youth who provided open-ended reflections about their perception of their relationships as emerging adults. This analysis could help to better understand relational experiences of this peculiar group, in the moment of their life in which they are out of foster family care (Greeson and Bowen, 2008).

This study addresses the question: what relational outcomes do Italian emancipated foster youth present during emerging adulthood?

## 2. Materials and methods

### 2.1. Participants

Participants were 26 Italian emancipated foster emerging adults (14 women and 12 men) between the ages of 19 and 25 (M = 22 SD = 2.89) with past experience of foster family, who were placed out of foster family at the age of 18 years, as required by the law (Table 1). They had an average age of 3.76 years at entry into social service care (range 0–8 years), although at their first placement in a foster family they were on average 6.7 years old. In almost all cases (92%) emancipated foster youth were placed in foster care and full-time (94%), and 19% of them experienced multiple placements.

**Table 1:** Socio-demographic characteristics of participants.

Id	Gender	Age at placement	Age at foster placement	Number of placements	Foster family composition	Number of other foster siblings	Placement type	Typology of foster care
1	Female	4	7	3	3	0	1	2
2	Male	3	4	2	2	1	1	2
3	Male	12	12	1	3	0	1	1
4	Male	3	4	1	3	0	1	2
5	Male	3	9	2	2	0	1	2
6	Female	5	7	2	2	0	1	2
7	Male	1	3	2	2	0	1	2
8	Male	5	5	1	2	0	1	2
9	Female	6	6	1	2	0	2	1
10	Female	6	13	2	3	0	1	2
11	Female	8	13	2	2	1	1	2
12	Male	7	16	3	2	0	1	2
13	Male	1	11	3	2	0	1	2
14	Male	6	6	2	3	0	1	2
15	Male	0	4	2	3	0	1	2
16	Male	1	3	2	2	0	1	2
17	Female	0	0	1	3	1	1	2
18	Female	5	6	1	1	0	1	2
19	Female	3	14	2	2	0	1	2
20	Male	0	2	2	2	0	1	2
21	Male	5	9	2	3	0	1	2
22	Male	2	2	1	1	1	1	2
23	Female	0	3	2	1	0	2	1
24	Female	0	0	1	3	0	1	2
25	Female	3	4	1	3	0	1	2
26	Female	10	12	1	3	0	1	1

Foster family composition: 1 = single foster parents (widower or divorced); 2 = biparental foster family; 3 = foster family with biological children. Placement Type: 1 = full time; 2 = part-time. Typology of foster care: 1 = voluntary foster care; 2 = court-ordered foster care.

They reported that their foster parents were mainly married (78%), had biological children (64%), and there was no other foster (85%) or adoptive (97%) children in the foster family. More foster parents (66%) met the biological family at the beginning of foster care.

### 2.2. Procedure

This study is part of a wider project, performed in a collaboration between the University and Social Services of the municipality of a medium-sized city in north-western Italy, focused on the outcomes of the family fostering experience from the perspective of youth (with such experience), foster families, and social workers. About this, a related work focused on the self-perception of young adults has been published recently (Bizzi et al., 2023). The sampling strategy resulted in a convenience sample to collect data on specific individuals (emancipated foster youth).

Recruitment was pursued through relevant gatekeepers, such as social workers, that facilitate recruitment by informing youths about the study and communicating contact information when youths were interested in participating and consented to have his or her contact details passed to researchers. Inclusion criteria were 18–25 years and having foster family experience. In total, 36 people were invited from social workers, but only 26 youth accepted and completed the study. Their reasons for not participating were lack of interest, difficulties with being audio-recorded, and time constraints. The participants were thanked for their time through a letter signed by the city’s mayor.

All youths were given the opportunity to ask questions before agreeing to participate, and it was made explicit that they could withdraw from the study at any time.

To reflect an authentic representation of the participants’ voices through the narration of their lived relational experiences, the present study employed a semi-structured and open-ended response interviewing procedure (Adams, 2015). These interviews were audiotaped and transcribed verbatim. All participants also completed a brief socio-demographic schedule. The entire session including the interview and the socio-demographic schedule last approximately an hour. All interviews were conducted by academic researchers in a private room in the office of the municipality. Researchers monitored risks related to emotional activation and offered the possibility of care or counseling after the interviews.

The ethical principles used to guide the conduct of this study were the guidelines set out by the Research Ethical Code of the Italian Association of Psychology. The procedures were approved by Department of Educational Sciences inside a specific agreement between Department of Educational Sciences and Local Social Services. Participation in the study was voluntary, and anonymity was assured using pseudonyms. The participants were informed that all data would be treated confidentially in compliance with Italian Law on Privacy n.196/2003 and GDPR 2016/679. Informed consent was obtained from all participants following a face-to-face explanation of the study.

### 2.3. Materials

An *ad hoc* socio-demographic schedule was built to investigate the following areas: data of the participant (e.g., age, sex); data on the foster family (e.g., family composition); data on the biological family (e.g., family composition); data on the foster project (e.g., history of placements, typology of foster care, etc.). In addition, a semi-structured interview was administered and audiotaped. The interview guide was designed to capture the information required from the study’s aim and was amended over the course of data collection according to emerging new topics. This approach enabled the researcher to ensure coverage of essential topics while still allowing the interview to be largely directed by the participant. The interviews started with the opening statement: “*I’m interested in exploring your experience of foster care. Perhaps we can begin with you talking about your family story*.” Following several open questions that investigated main areas: (1) relationship with the biological family; (2) relationship with the foster family; (3) relationship with the partner and peers. Questions were designed to be open-ended in nature to elicit reflection on the experience of foster care. An example of open question of this interview was “*Could you describe your relationships with your biological family/foster family/ in the past/during the adolescence/in the present*?”

### 2.4. Data analysis

Foster care experiences are best explored using qualitative inquiry for its ability to capture richness and complexity (Schwab and Syed, 2015). A qualitative thematic analysis was selected for the present study (Alholjailan, 2012; Kiger and Varpio, 2020). We chose Grounded Theory as an adopted approach to inform the data collection, data analysis (coding procedures) and thematic analysis. We selected a constructivist grounded theory approach to emphasize the power of narratives (Charmaz, 2016). Emancipated foster youth’ voices are underrepresented in Italy, and constructivist grounded theory could create a space to generate a meaningful reading comprehending the subjectivity of their experience (Charmaz, 2017).

Codes were derived directly from segments of data, and these codes were used to sort and develop an understanding of what was happening in the social situation being studied (Charmaz, 2006). All concepts generated from initial open coding have been identified in a smaller subset of thematic categories via axial coding (Corbin and Strauss, 1990), so core characteristics of the interview data were captured as a taxonomy of codes. According to (Merriam, 2009), reliability in coding represents a subjective consensus in a specific research team, at a specific time, and not an absolute truth that exists outside of the data. Two coders (FB and PC) assessed the reliability of the coding taxonomy using randomly selected narrative samples from 20% of the data (McLean and Pratt, 2006). Raters were trained to reach the acceptable levels of agreements of .80 (Cicchetti, 1994; Syed and Nelson, 2015). Then the two researchers privately and independently coded narratives using a codebook and the taxonomy for emerging themes or recurring domains of meanings across the narratives (Lofland and Lofland, 1995; Rossman and Raillis, 1998). A reflexivity-based iterative process was undertaken between the researchers and the different background from clinical and social psychology was used as a resource to better interpret the content and the meaning of the texts (Corbin and Strauss, 1990). All disagreements were discussed, and a code was agreed upon. Based on the initial codes, core categories were developed using focused coding. Using the constant comparative method (Glaser and Strauss, 1967), existing data were compared with new incoming data for similarities, differences, and variation of meaning. The coding manual was updated during the process. Finally, social workers and foster youth were asked to share the results.

NVivo version 12 was used to manage the data and to organize nodes into higher-order codes and categories generating themes based on them.

## 3. Results

The results show the presence of key concepts expressed by emancipated foster youth, which can be categorized into relational dimensions such as “Feeling supported,” “Sense of belonging,” “Good memories,” “Relationship with family of choice,” “Staying in touch,” “Devaluation,” “Refuse,” “Bad memories,” and “Isolation.” These categories are organized in two themes: the first pertains to positive relational outcomes and includes dimensions that provide safety and stable identification for emancipated foster youth (“Feeling supported,” “Sense of belonging,”” Good memories,”” Relationship with family of choice,” “Staying in touch”) in Table 2, who often experience instability and uncertainty, as we delineated above. The second theme, termed negative relational outcomes includes cognition, behaviors, and experience that can undermine and break relational ties: “Devaluation,” “Refuse,” “Bad memories,” and “Isolation” in Table 3. In the subsequent sections, we will discuss these themes and their meanings in greater detail. This classification underscores the multifaceted nature of the foster care experience for many young adults, incorporating both positive and negative relational aspects.

**Table 2:** Positive relational outcomes: categories and subcategories.

Feeling supported	Sense of belonging	Good memories	Relationship with family of choice	Staying in touch
Social support from foster family	Foster parent’s identification as own family	Possibility to identify positive figures in the family of origin	Partner perceived as a savior	Continuity in the relationship with biological parents
Social support from partner and his/her family	Sense of home	Positive attributions to biological parents	Early couple relationship	Continuity in the relationship with foster parents
	Acceptance from partner’s family	Positive attributions to foster parents	Parenthood as an accomplishment	Presence of foster parents in important life events
	Welcoming of the local community			Positive relationship with peers

**Table 3:** Negative relational outcomes: categories and subcategories.

Devaluation	Feeling of being rejected	Bad memories	Isolation
Devaluation of biological parents	Lack of interest from the biological family	Maltreatment	Absence (physical and psychological) of the biological family
Devaluation of foster parents	Non-acceptance from the biological children of the foster family	Role reversal with biological parent	Lack of figures to rely on
	Desire to proceed with adoption		Absence of contact with the foster family
			Need for loneliness

### 3.1. Positive relational outcomes

#### 3.1.1. Feeling supported

The perception of feeling supported is evident for many participants in this study. The main source of social support is represented for these young people by the foster family, from which they feel supported in the present from an emotional point of view but also for their physical presence, as one-woman reports:

There is extreme trust, an absolute presence. Their support is not comparable to that of anyone else. They give me a lot of support in everything I do (B, woman, 21 years).

This social support from the foster family is rooted in a supportive relationship from the beginning of the assignment and seems to also concern the possibility of feeling supported in future projects. A youth expresses this as:

They were very close to me, even in the realization of my subsequent dreams. And above all, in the very first period of the custody, they supported me even economically. I must say that they have never let me miss anything (G, man, 19 years).

Another significant source of support is the partner, which represents an important point of reference with which to confide, as emerges from the words of this youth:

When I have something wrong, I talk to her anyway. I’m very confident with her (M, man, 23 years).

Furthermore, also the partner’s family frequently supports these emerging adults, as one participant stated:

Both he and his family have always been present and supported me since the first moment (J, woman, 22 years).

Therefore, in participants’ narratives, the feeling of support refers to a vertical temporal dimension, which recognizes the relational experience with significant people (foster parents or partner) along the time, from the beginning of foster care to the present moment, as a positive outcome for these youth.

#### 3.1.2. Sense of belonging

The sense of belonging refers to a sense of confidence that one has as a group member regarding acceptance from the group. This sense of belonging could be used to evaluate the psychological connection between emerging adults and different contexts of their life; indeed, this membership is associated with the foster family, the partner’s family, and the local community. For most participants in our study, foster placements are considered essentially quasi-adoptive in nature, and the youth view their foster carers as if they are their biological parents in the past and in the present, as some participants state:

It was certainly a unique and fundamental experience, truly like a mother (M, man, 19 years); They are my family, that is my foster parents, who raised me (C, woman, 21 years).

The foster parent’s identification as a family to belong is very frequent for participants, who express this membership by calling them “mum” and “dad” as this youth states:

I trusted them as if they were my parents from the first day. Once they told me, ‘If you want you can call us mom and dad,’ and since that day I’ve done it! (A, man, 23 years).

This makes young people feel a sense of home that includes feeling of connection to the place where they lived for many times with foster parents:

When talking about home, family, it was the location of my foster parents (A, woman, 22 years); It‘s my family. I feel at home. I’m like any normal person who lived with a normal family (B, woman, 21 years).

In our participants’ words home is where they feel that their roots are planted, and they identify this place in foster family, not in biological one.

Various relationship experiences are mentioned by participants to describe their sense of belonging to a relational context and the positive emotions that could arise from these experiences. Also, acceptance from a partner’s family plays an important role in the development of this sense of belonging because it helps to make people feel accepted and welcomed:

[My partner’s parents] never judged me, never criticized me. They always accepted me as a daughter (J, woman, 22 years).

Finally, a welcoming community contributes to developing the sense of belonging of our participants because it represents a location in which newcomers could feel valued and their needs could be served. The neighborhood represents a source of well-being; indeed, these positive social contexts may affect the sense of belonging to a place, that could be very important for foster youth that lived in more homes.

It’s a place where they welcomed us, both me and my mother and my brother. They welcomed us very well (B, woman, 21 years).

The sense of belonging develops within an ecological framework, in which the fit between a person and the environment (that includes micro and macro systems) can affect the person’s feelings in a positive way.

#### 3.1.3. Good memories

The sub-theme good memories summarize participants’ recollections of their past experiences. Some statements refer to the possibility to identify positive figures in the family of origin, as this youth states:

Up to four and a half years, I was with my grandmother who gave me a little bit of a mother. She taught me everything (E, man, 24 years).

For most participants, these positive relational figures are grandmothers and grandfathers. A young man expresses this as:

The relationship with my grandmother was, I think, the best relationship in my life. I have only beautiful memories (J, man, 23 years).

Youth also remember positive moments lived with biological parents and know how to find positive attributions to biological parents, as indicated by these sentences:

When I was little, my father always tried to cheer me up when I cried (E, man, 24 years); Mom always bent over backward to get things done when I was a child (A, man, 23 years).

The category “Good memories” also includes positive attributions to foster parents, as one young woman said:

I found a family that, despite some flaws from me and from them, they always loved me from the first moment they saw me (B, woman, 21 years).

Good memories represent some light in the dark experience of foster care because our participants identify positive behaviors and positive figures in their past. This possibility could be a resource also in their present because they seem able to underline the positive elements of their relational experience.

#### 3.1.4. Relationship with family of choice

The relationship with family of choice sub-theme describes the situation of youth who have built a family of choice and who are happy about it. An idea that is frequently brought up by our participants is that the partner could be perceived as a savior, as this young man states:

I think that one of the biggest rescues that can come is a fiancée, boyfriend, or otherwise someone who doesn’t let you think about everything around you […] she changed my life completely. She was the start up that I needed (M, man, 23 years).

These important romantic relationships often started when participants were very young:

At 16 I got engaged to my current husband (C, woman, 25 years).

Participants speak about premature engagement or marriage, as this youth woman states:

I wanted to marry so young because I still think that A. is the most important person in my life (J, woman, 22 years).

In female narratives is present also the idea that parenthood is an accomplishment in women’s’ life. Participants narrate about the experience of motherhood and the relationship with the baby as rewarding:

[My child] fills my life … And what can I say, this makes me really happy in this moment of my life (J, woman, 22 years).

This idea emerges only in female narratives. The building of a stable couple relationship and the engagement in a parental project, despite they experienced a fracture in the bonds with the family of origin during childhood, represents a positive outcome for these adults who seem to have “saved” the ability to build ties.

#### 3.1.5. Staying in touch

The perception of staying in touch is another emergent finding in our work. Participants describe the continuity in the relationship with biological parents, which concerns their past experience in foster care, as this young woman states:

One Sunday, I went with my mother; one Sunday, I went with my dad. Sometimes they came to pick me up at school. I always saw my parents (C, woman, 25 years).

This also appears in the present experience and outlines the possibility for these youth to have contact with their biological family over the years:

Now I see them. Every now and then I go to their house. They live nearby, and we call each other anyway (G, man, 21 years).

Youth also remember continuity in the relationship with foster parents, which concerns their current activities, as this young man states:

Last year I decided to take the season ticket too, so with my [foster father] we’ll see each other on Sunday for the match (G, man, 19 years).

Another element that contributes to the perception of being in contact is the presence of foster parents in important life events, as this young woman reports:

Mum (foster mother) acted as my wedding witness (C, woman, 25 years).

Many participants report their satisfaction for their positive relationships with peers, as emerges from the words of this young woman:

I have many different groups of friends. Just so different. With a group, for example, we like to stay at home, play games or watch movies together. With another group of friends, who are those who are a little closer, we make trips, or in general we do aperitifs or, in any case, things that are done every day that everyone does (B, woman, 21 years).

The possibility to have a lot of friends seems to be associated with our participants with their personality traits, as these participants states:

I have many friends because I have this very expansive character (M, man, 20 years); I am a very sunny person, I make friends right away (A, man, 20 years).

The possibility staying in touch refers to the relationships that the participants maintain both inside and outside the family. This aspect could contribute to the building of a positive self-image.

### 3.2. Negative relational outcomes

#### 3.2.1. Devaluation

From a relational point of view, participants frequently made use of devaluation by attributing negative characteristics to others. This mechanism suggests the need to use defences against anxiety that derives from essentially ambivalent aspects in the perception of oneself and others. These devaluations concern the biological family whose positive aspects are completely erased, as aroused by the words of this youth:

I completely removed them from my life, so there is no positive side because they behaved badly. They behaved badly with us because they left us alone, so although there were meetings with them, I wanted to remove them from my life (R, man, 23 years).

However, in the narratives is also present a devaluation of foster family, which includes disapproval of the family members, as this young woman states:

The thing that still made me angrier is that once I asked her if she didn’t even feel a little bit ashamed, and she replied that she had a clear conscience and that she did everything she could do. She is not really a civilized person (R, woman, 19 years).

Participants seem to use devaluation to address feelings of uncertainty in foster care. This concerns both relationships in biological and foster families.

#### 3.2.2. Feeling of being rejected

Another significant area concerns the feeling of being rejected, which is the feeling of not having been accepted and desired by the people in one’s life.

This emotion seems to be rooted in a perception of disinterest from the biological family, as this youth reports:

I have never seen her again. The last time I saw her, I was six years old, and I was in the Institute (P, man, 19 years).

Another important element is the experience of a lack of acceptance in the foster family. There is a lack of acceptance by the biological child of foster parents, which underlines the importance of thinking about foster care as a device that involves the whole family and not just the parental couple, as this young woman states:

They were ready as a couple, but they were not ready as a family in the sense that maybe they were ready because they had only a child … He was an only child, so they were ready for such experiences, but actually [my child] never accepted that I entered their family (J, woman, 22 years).

A relational suffering that arises from narratives is about participants’ awareness that the foster parents desired to proceed with adoption and therefore that they were not the first choice:

They told me they wanted to adopt a boy. They tried in every way to meet and to accept, but they even told me about children they had known before me (S, man, 23 years).

The perception of being rejected represents a negative outcome from a relational point of view because if one is not accepted by others one can feel negative emotions and this might also alter the way one views one’s life.

#### 3.2.3. Bad memories

Bad memories include the narratives that the participants report with respect to past experiences that left a mark. They refer to maltreatment, as this young woman states:

My mother’s companion tried several times to have acted on me, touching me (P, woman, 24 years).

However, this category is present only in female narratives. Bad memories include also situations in which the participants have to take on heavy situations by performing a role reversal with the biological parent not feeling cared for by them:

She (the mother) cannot, I looked after her, I liked when she was at home, but she cannot be of any support (S, woman, 22 years).

#### 3.2.4. Isolation

Finally, the last area relating to negative relational outcomes concerns isolation. There is a shared experience among participants of feeling isolated and like an outsider. This feeling derives from having experienced the absence of the family of origin:

I haven’t seen her for a while. I haven’t seen her for 10 years, even more (P, man, 19 years).

but also, from not having in the past or in the present figures to rely on, someone who take care of them, as this young woman states about herself and her brothers:

It was our responsibility to make things. We had to eat out because we were too little, and we didn’t know how to cook (S, woman, 22 years).

The isolation also concerns the breakdown of relationships. In particular, the absence of contact with the foster family in the present emerges, for instance in the words of this young man:

I closed relations immediately afterward. As soon as I left the family, I closed the relationship. They never reopened (R, man, 23 years).

Finally, there sometimes emerge the need to withdraw from the social context and from social relations and to experience the need for loneliness, as this young woman states:

I asked to be put in an office alone, to work alone in a more serene way (S, woman, 22 years).

Isolation seems to be an experience that covers different aspects of the participants’ life.

## 4. Discussion and conclusion

The present study represents the first study in Italy that investigates the relational outcomes in a group of emancipated foster youth. It allows us to reflect *a posteriori* on the experience of family custody and on the possible outcomes reported by young people, not only in relation to academic and work success, but with regard to relational outcomes.

The findings show the complexity of the path of emerging adulthood, highlighting the diversity of experiences that emancipated foster youth perceived about their relationships. From participants’ narratives emerge two different themes: positive relational outcomes and negative relational outcomes (Greeson and Bowen, 2008) that often occur simultaneously in participants’ narration, outlining a complex and sometimes ambivalent experience.

Research suggests that social connectedness and healthy relationship is protective factors against many forms of aversive childhood events (Ludy-Dobson and Perry, 2010). From our work emerge some positive relational outcomes recognized from participants (“Feeling supported,” “Sense of belonging,” “Good memories,” “Relationship with family of choice,” and “Staying in touch”) related to satisfaction about their lives. In line with Wissö et al. (2019), this study points out that the sense of belonging is a positive outcome for individuals in foster care due to the typical instability of family relationships. A sense of belonging is associated with meaning in life (Lambert et al., 2013) and this could represent a positive outcome, for people who have unexpected and uncommon experiences, such as family custody. Another positive relational outcome for individuals in foster care is the good memories about foster and biological parents. Although foster individuals can experience their biological parents as more vulnerable and trust them less than their foster parents, which is comprehensible considering the problematic background and living conditions of most parents, positive attributions of both parents are common in this study.

These ideas coexist with their exposition to loss, separation, and rejection experiences (Sulimani-Aidan and Melkman, 2018; Driscoll, 2019). Considering the negative outcome of foster care, in line with the literature (Fabricius and Hall, 2000; Finley and Schwartz, 2004; Rohner et al., 2005), emerges the feeling of having been rejected by the biological parents (but also from other family members), the perception of disinterest from the biological family, the sense of regret about foster parents’ desires to proceed with the adoption and the sense of dissatisfaction for the relationship with the biological and foster parent. These themes could represent some key elements of troubled ruminations about foster parents conceptualized by Schwartz and Finley (2010) as a critical model of functioning, particularly in emerging adulthood. In line with this theory, these negative relational outcomes about biological, but also foster parents made up of devaluation, feeling of having been rejected and bad memories about infancy could reflect a perceived family rejection and may represent a form of distress impairing psychosocial and relational functioning in emerging adulthood (Schwartz and Finley, 2010). These findings reveal evidence that positive and negative views of their significant relationships coexisted, making complex and sometimes ambiguous relational experiences. In this sense, there is a need of integration and foster family experience may help to facilitate this process. In addition, the idea that this perception of having been rejected could concern the relationships between brothers, and between the foster children and the biological children, may help the social works to think about foster care as a device that involves the whole foster family and not just the foster parental couple. Previous searches underline that fosters siblings do not feel that they are perceived as part of the foster team (Raineri et al., 2018).

Turning on positive outcome, we must account for the fact that the development of a relationship with sensitive and responsive foster parents enriches opportunities of foster individuals (Maaskant et al., 2015), empowers the capacity of a foster child or adolescent to reflect upon and understand the mental state of oneself and others, enabling them to understand the difficulties of biological parents. Healthy relationships make emancipated foster youth better able to regulate emotional distress, and subsequently heightens their psychosocial development and resilience (Stein, 2006). Recent study (Ball et al., 2021) underlines that positive experiences in relationships with foster parents and social workers may lead to lasting and supportive relationships at the transition to adulthood. Furthermore, stable couple relationships in emerging adulthood are considered as frequent outcome for individuals with a secure attachment style (Fagundes and Schindler, 2012) and it might continue to predict security through the life (Sutton, 2019). Moreover, the experience of current good relationships with peers is related to a positive self-image as people with good social competence. This can provide a core form of support for their identity. Likewise, the community involvement represents a potential benefit for emancipated foster youth; some studies (e.g., Magson et al., 2016) highlight that the feeling of belonging to the community could decrease risk-taking behaviors among disadvantaged emerging adults. Sarriera and Bedin (2015) developed a socio-community well-being model, including variables related to the feeling of belonging to the community in the idea of well-being. Therefore, an increase in relational satisfaction in life requires interpersonal and collective spheres to be more integrated (Prillelstensky, 2004). In this sense, resilience is not only regarded as a by-product of exposure to moderate adversity and protects individuals against maladjustment with future stress, but also offers protective factors, such as the ability to maintain a close relationship with other capable adults, emotional regulation, and acceptance of their own history that counteract the potential risks and vulnerabilities (Yates and Grey, 2012).

Moving on negative relational outcome, it is interesting to note that the results of category bad memories underline a gender difference about maltreatment recollection because this memory is reported only by women. This result is in line with previous research that underlines how women have often been considered to be the predominant victims (Cardinali et al., 2018), but this could also be associated with men’s difficulties to report these experiences in the Italian cultural and social context where for a man, reporting intimate partner violence behavior might induce shame, guilt, and embarrassment, which possibly decreases the likelihood of disclosure of such violence (Felson and Paré, 2005).

Comparing these findings with the foster care literature (Cooley et al., 2018) in which mainly negative outcome such as difficulties in developing and maintaining trusting and supportive relationships in adulthood emerge, this study confirms the existence of a deprivation theme that appears to be pervasive throughout life and not only limited to childhood. However, the theme “need for loneliness” adds to the negative outcomes an innovative aspect whereby emerging adults who are discouraged by negative experiences do not seem to have enough resources to deal with the community. Although some of the themes found in the current study mirror the findings from the literature on emerging adulthood like the importance in this critical transition of developing good relationships, novel ideas sprang from our interviews like the supporting role of partners. These complement the literature that stressed the importance of social capital (Hook and Courtney, 2011; Skobba et al., 2018), that is also confirmed in our work highlighting the importance of peers and community involvement. This additional support received not only from biological and foster families, could promote the strengthening of identity and the reduction of feelings of loss and rejection (Delgado et al., 2019; Wissö et al., 2019). Romantic relationships become a central theme in the transition to adulthood (Oudekerk et al., 2015), and even though the early relationships in the biological family, these youth seem to develop some ability to manage autonomy and related challenges in adult relationships across different social domains (romantic relations, friendship, family). With respect to the emotional themes, it is important to note that narratives about the transition to parenthood occurred only in women’s interviews and this could be coherent with the “mother-centered” ideal highlighted from different studies in Italy (Bertolini et al., 2015; Wiß and Greve, 2019) and reflect the idea that motherhood represents a key relational element of female identity. However, it’s important consider that male participants have not yet experienced paternity. Emerging adulthood provides youth with opportunities to reflect on their past experiences in biological and foster families and to look forward to the present and future (Arnett, 2004, 2015; Schwartz et al., 2011). This process is more complex due to foster experience.

The present study represents the first qualitative research on emancipated foster youth in Italy exploring their relational outcomes in emerging adulthood. This is a particularly relevant aspect in the Italian contest since the Service System does not follow up on the path of young people released from foster care and therefore can also be useful for practitioners to better understand how relational aspects are important to consider. Longitudinal studies that track youth relational trajectories over time would provide valuable insights into the challenges they face and the support they may need beyond the immediate emancipation period. Furthermore, this study’s strengths include the breadth of the group of participants, representing a population that is difficult to reach and poorly represented in previous studies (Miller and Collins-Camargo, 2016). However, this study provides youth perspective, but consequently, other perspectives are lacking, and they could be relevant to a more complex understanding of the relational outcomes. Future research could consider incorporating other perspectives, such as those of foster parents, social workers, or biological families to gain a more holistic understanding. The foster care placements of the study sample appear impressively stable despite national data suggesting a major frequency of multiple placements in the history of emancipated foster youth (only 4,5% has not a history of multiple placements; Minister for Labour and Social Policy, 2021). Therefore, this may suggest a more favorable psychological condition of participants included in the study. Consequently, the stability of foster care placements observed in our participants may not be reflective of the experiences of all emancipated foster youth, as national data suggest a higher prevalence of multiple placements. Future studies could aim for larger and more diverse groups of participants to improve the representativeness of the findings. Furthermore, we did not consider the number and the type of adverse childhood experiences they had and how they impact their current relational health. Future research could explore the relationship between ACEs, relational outcomes, and the long-term effects of trauma on emancipated foster youth. Another important aspect that should be acknowledged as a potential area for future research is the lack of consideration for gender differences among emancipated foster youth. Exploring how gender intersects with relational outcomes could provide valuable insights into the unique needs and experiences of male and female Italian emancipated foster youth. Future studies could adopt a gender-sensitive approach, examining how gender influences youth relationships, support networks, and overall well-being. Research could also investigate whether gender plays a role in the formation of bonds and the establishment of a sense of security with foster parents. For example, it might explore whether male or female youth have different expectations or preferences regarding the gender of their foster parents, and how these expectations might influence their perceptions of support and acceptance. A further limitation of this study could be the difficulty of integrating distant perceptions and emotions concerning relational outcomes to reflect on their own level of satisfaction.

Professionals could work on relational outcomes and memories, especially during a critical transition like emerging adulthood, to support youths in making sense about their past experience. It appears very important to promote the formation of social workers who are competent in the analysis of relational outcomes and in working to promote positive relationships for their protective function in the future of these youths. The use of narrative to reconstruct auto-biographical memory could serve to make sense of one’s past, present, and anticipated future (McLean, 2005), supporting and meeting challenges of this special transition to adulthood.

## Data availability statement

The raw data supporting the conclusions of this article will be made available by the authors, without undue reservation.

## Ethics statement

The studies involving human participants were reviewed and approved by the Department of Education under a special agreement between the Department of Education and local social services. The ethical principles for conducting this study were the guidelines of the Ethical Research Code of the Italian Association of Psychology. The patients/participants provided their written informed consent to participate in this study.

## Author contributions

FB and PC contributed to analysis, interpretation of data, and drafted the manuscript. LM contributed to conception, design, acquisition, and recruitment of data. All authors critically revised the manuscript and gave final approval. All authors contributed to the article and approved the submitted version.

## Funding

This work was supported by Municipality of Genova.

## Conflict of interest

The authors declare that the research was conducted in the absence of any commercial or financial relationships that could be construed as a potential conflict of interest.

## Publisher’s note

All claims expressed in this article are solely those of the authors and do not necessarily represent those of their affiliated organizations, or those of the publisher, the editors and the reviewers. Any product that may be evaluated in this article, or claim that may be made by its manufacturer, is not guaranteed or endorsed by the publisher.
